# Nineteen years retrospective analysis of epidemiology, antifungal resistance and a nomogram model for 30-day mortality in nosocomial candidemia patients

**DOI:** 10.3389/fcimb.2025.1504866

**Published:** 2025-02-03

**Authors:** Zhang Dai, Xuhong Lan, Minjing Cai, Yunhui Liao, Jingwen Zhang, Naifang Ye, Xinxin Lu, Jiajia Wang, Yun Xiao, Yan Zhang, Yihui Yao, Xianming Liang

**Affiliations:** ^1^ Centre of Clinical Laboratory, Zhongshan Hospital of Xiamen University, School of Medicine, Xiamen University, Xiamen, China; ^2^ Department of Clinical Laboratory, Xiamen Hospital of Traditional Chinese Medicine, Beijing University of Chinese Medicine, Xiamen, China; ^3^ Department of Clinical Laboratory Medicine, The Second Hospital of Anhui Medical University, Anhui Medical University, Hefei, China

**Keywords:** candidemia, *Candida albicans*, resistance rate, mortality, nomogram

## Abstract

**Background:**

The incidence of nosocomial candidemia has increased in recently years, however, the epidemiological data remain insufficient in China.

**Methods:**

A total of 234 candidemia patients were included from Xiamen University Zhong Shan hospital between January 2006 and October 2024. Incidence, species proportion, distribution, antifungal drug resistance of candidemia was analyzed. A nomogram model for 30-day morbidity of candidemia was determined using the least absolute shrinkage and logistic regression analysis.

**Results:**

The incidence of candidemia increased in recent years (2020: 0.025%, 2021: 0.029%, 2023:0.022%). The dominant species of candidemia were *Candida albicans* (n=99,42.31%), *Candida parapsilosis* (n=47,20.09%), *Candida tropicalis* (n=43,18.38%), *Candida glabrata* (n=31,13.25%). Departments with a higher detection of candidemia included intensive care unit (n=55), emergency department (n=24) and hepatobiliary surgery (n=22). *Candida tropicalis* performed the highest resistance to azole (fluconazole: 55.81%, voriconazole:55.00% and itraconazole:58.14%). The resistance of *Candida albicans* to fluconazole, voriconazole and itraconazole were 32.32%, 23.53% and 31.31%. The mortality rate of 30-day discharge for candidemia reached 52.99%. 205 cases of candidemia patients from January 2006 to December 2023 were included as the training set, while 29 cases of candidiasis patients from January to October 2004 were included as the validation set. Five independent factors included *Candida albicans*, decreased albumin, multiple organ dysfunction syndrome, solid tumor and septic shock were adopted in a nomogram for 30-days mortality of candidemia. In the training set, the area under curve was 0.866 (95%CI: 0.817-0.916), the optimal cutoff value was 0.617, the sensitivity was 80% and the specificity was 80.4%. In the validation set, the area under curve was 0.808 (95%CI:0.737-0.970), the optimal cutoff value was 0.543. The sensitivity was 72.7% and the specificity was 83.3%.

**Conclusion:**

The incidence of nosocomial candidemia has risen in recent years. *Candida albicans* remains the primary species, with the highest incidence is intensive care unit. *Candida tropicalis* exhibits the highest resistance rate to azole drugs. A nomogram predicting 30-day mortality discharge for candidemia patients has been constructed, and the independent risk factors including *Candida albicans*, multiple organ dysfunction syndrome, septic shock, solid tumors, and decreased albumin.

## 1 Introduction

Candidemia, a bloodstream infection caused by *Candida* species, has become increasingly prevalent in recent years and poses significant challenges to healthcare systems (Ré et al., 2021; Ferrando and Castagnola, 2023). The incidence and mortality of candidemia show great variability due to differences in medical levels and health conditions among different regions (Yang et al., 2021; Bilal et al., 2023; Kitaya et al., 2023). Candidemia greatly complicates the treatment process, leading to prolonged hospital stays and increased budget expenditures (Zeng et al., 2020). Futhermore, it significantly increases the length of hospital stays due to the need for prolonged antifungal therapy, management of organ dysfunction, and complications from the infection itself (Klingspor and Hällgren, 2024).

Currently, more than 20 *Candida* species have been identified as potential causes of candidemia.*Candida albicans* is the main pathogen of candidemia, accounting for over 50% of candidemia, however, *non-Candida albicans* species of candidemia have been increasing in recent years (Rola et al., 2023). The resistance rates of *Candida speices* causing candidemian to antifungal agents are increasing year by year, particularly to azole drugs.The resistance rate of *Candida species* such as *Candida tropicalis* and *Candida glabrata* to fluconazole are closely linked to the overuse of antifungal medications in hospitals and communities, which has resulted in the development of resistant strains and complicated the effective treatment of candidemia (Meng et al., 2020). In particular, the increase in cases of COVID-19-related candidemia in recent years has further exposed the accelerating trend of antifungal resistance (Najafzadeh et al., 2024).

The high mortality rate in candidemia is driven by several factors, including advanced age, immunosuppressive conditions, and co-morbidities such as organ dysfunction (Poissy et al., 2020). Conditions such as gastrointestinal disease, liver cirrhosis, malignancies, and renal failure requiring dialysis are associated with higher mortality rates in candidemia patients (Bartoletti et al., 2020). The risk factors for mortality due to candidemia will change significantly over time and across different geographic regions. The study of Kahan Y et al. found that the attributable mortality rate of candidemia in pediatrics was about 28.5% (Kahan et al., 2023). Overall mortality from candidemia has been reported in multiple studies to be 30%-50%, which is associated with disease severity, delayed diagnosis, and inappropriate antifungal therapy (Mazi et al., 2022). A study from intensive care unit in China reported a 51.2% all-cause mortality rate for candidemia, identifying early physiological indicators like the Glasgow Coma Scale (GCS) score and PaO2/FiO2 ratio as independent mortality risk factors (Xiao et al., 2019), while a European meta-analysis found a 30-day mortality rate of 37% in intensive care unit patients and 38% in general teaching hospital patients. Delays in antifungal therapy, lack of early catheter removal, non-*Candida albicans* infections, and severe comorbidities are key risk factors for candidemia mortality (Koehler et al., 2019). Poissy et al. reported that delays in antifungal therapy, lack of early catheter removal, ICU setting, non-*Candida albicans* infections, and severe comorbidities are key risk factors for candidemia mortality (Keighley et al., 2020). Identifying the risk factors leading to mortality from candidemia is crucial for early intervention and reducing the mortality rate.

This retrospective study aims to evaluate the incidence, distribution of departments, antifungal resistance of *Candida* species and construct a nomogram model for 30-days mortality risk factors of candidemia in Xiamen, Fujian, China over past 19 years. The outcome of this study will assist the clinicans in managing nosocomial candidiemia in the local region.

## 2 Materials and methods

### 2.1 Study design and participants

This study performed a single-center retrospective analysis of candidemia at Xiamen University Zhongshan Hospital, which provides health services to 4.5 million people in Fujian province, China. Candidemia was defined as the presence of at least one positive blood culture for *Candida* obtained from peripheral or central bloodlines, accompanied by clinical signs and symptoms indicative of a *Candida* infection. The diagnostic criteria for candidemia were based on the guidelines for the revised definition of invasive fungal disease (IFD) from the European Organizations for the Research and Treatment of Cancer/Mycoses Study Group (EORTC/MSG) (Donnelly et al., 2019).

The demographic and clinical characteristics were based on electronic medical records and laboratory information systems of inpatients at our hospital from January 2006 to October 2024. A total of 325 patients have been diagnosed with candidemia. Among them, 13 patients less than 18 years old, 22 patients with incomplete medical history data, 18 patients with community-acquired infections before hospitalization, and 38 patients with mixed infections from other yeast-like fungi or bacteria were excluded, resulting in a total of 234 cases that were included in this study.

The hospital’s Human Research Ethics Committee approved the study by the Declaration of Helsinki guidelines (reference number: xmzsyyky 2024-020).

### 2.2 Data collection

The demographic data and clinical characteristics of participants were from hospital information system (HIS), electronic medical records (EMR), laboratory information management system (LIS). The data were analyzed based on the patients’ age, sex, admission ward, underlying comorbidities, fungal drug resistance testing and invasive procedure. The analysis involved the results of laboratory biomarkers, including blood test, hepatic and renal function, and inflammation markers. This study obtained information on patients’ survival up to 30 days after discharge by conducting telephone follow-up.

### 2.3 Candida isolation, identification, and susceptibility testing

Blood cultures were collected during the onset of chills or fever, preferably before the administration of antibiotics. A minimum of two sets of blood cultures were obtained on each occasion, with each set consisting of one aerobic bottle, one anaerobic bottle, or one fungal blood culture bottle. To reduce the risk of contamination, each set was drawn from distinct puncture sites. If necessary, two or more blood cultures were performed within a 24-hour period to assist with diagnosis. For adults, the recommended blood volume per bottle was 8 to 10 milliliters. All blood culture collection procedures strictly adhered to aseptic techniques to maintain sample integrity. The collected samples were delivered to the laboratory within one hour and promptly incubated using an automated blood culture system (De Plato et al., 2019). The blood samples were cultivated in a fully automated blood culture system (BACT/ALERT-3D BioMérieux, France before 2006 to 2016 and BACTEC™ FX, American 2016 to 2024). Blood samples from positive bottle were subjected to microscopy with gram staining and culturing on CHROMagar-Candida and Sabouraud dextrose agar plates (Autobio Co Ltd). MALDI-TOF MS identification was performed on a Microflex LT/SH (Bruker Daltonik, Bremen, Germany) using the MBT Compass IVD 4.2 (Build 100). All *Candida* species before 2024 were re-identified using MALDI-TOF MS in January 2024.

The ATB FUNGUS 3 kit (bioMérieux, France) was used to assess the antifungal drug resistance of four drugs (amphotericin B, fluconazole, itraconazole, and voriconazole) according to the manufacturer’s specifications. The study utilized the official standards of the Clinical and Laboratory Standards Institute (CLSI) in the United States (Weinstein and Lewis, 2020), the European Committee on Antimicrobial Susceptibility Testing (EUCAST), and the U.S. Food and Drug Administration (FDA) to determine drug resistance to determine the breakpoint of antifungal agents (Wiederhold, 2021). All the CPBs for drug resistance were listed in **Table 1**. The quality control strain of *Candida albicans* (ATCC 22019) and *Candida krusei* (ATCC 6258) were applied. According to the Clinical Breakpoints (CBPs) of antifungal drugs, *Candida* species were classified into sensitive (S), intermediate (I), susceptible dose-dependent(SDD) and resistant (R) categories based on the minimum inhibitory concentration (MIC). The resistance of *Candida glabrata* to voriconazole and itraconazole were used to wild-type and non-wild-type analyze drug resistance. All of *Candida* species before 2024 were retested for drug resistance according to the above criteria in January 2024.

**Table 1:** Standards for the clinical breakpoints in resistance to antifungal drugs based on the minimum inhibitory concentration.

Antifungal agents	Candidemia species	CBP version	R^a^>=	I^b^	SDD^c^	S^d^<=	WT/non-WT^e^
Amphotericin B	*Candida albicans*	EUCAST2020	1			1	
	*Candida parapsilosis*	EUCAST2020	1			1	
	*Candida tropicalis*	EUCAST2020	1			1	
	*Candida glabrata*	EUCAST2020	1			1	
Fluconazole	*Candida albicans*	CLSI 2019	8		4	2	
	*Candida parapsilosis*	CLSI 2019	8		4	2	
	*Candida tropicalis*	CLSI 2019	8		4	2	
	*Candida glabrata*	CLSI 2019	64		<=32		
Voriconazole	*Candida albicans*	CLSI 2019	1	0.25-0.5		0.12	
	*Candida parapsilosis*	CLSI 2019	1	0.25-0.5		0.12	
	*Candida tropicalis*	CLSI 2019	1	0.25-0.5		0.12	
	*Candida glabrata*	CLSI 2019					0.25 ECV ^f^
Itraconazole	*Candida albicans*	FDA	1	0.25-0.5		0.125	
	*Candida parapsilosis*	EUCAST2020	0.125			0.125	
	*Candida tropicalis*	EUCAST2020	0.125			0.125	
	*Candida glabrata*	CLSI 2019					4 ECV

^a^Resistance; ^b^Intermediate; ^c^Susceptible dose-dependent; ^d^Susceptible; ^e^Wide type/non-wild type.

^f^The current evidence is insufficient to establish a correlation between the *in vitro* sensitivity results of *Candida glabrata* against Voriconazole and its clinical efficacy.

CBP, the clinical breakpoints; EUCAST, European Committee on Antimicrobial Susceptibility Testing.

CLSI, Clinical and Laboratory Standards Institute (CLSI) in the United States; FDA, U.S. Food and Drug Administration; ECV, Epidemiological cutoff values.

### 2.4 Statistical analysis

All data were analyzed using SPPS26.0 statistical software (IBM SPSS Statistics, IBM Corporation, Armonk, NY, United States) and R software (version 4.2.0; R Foundation for Statistical Computing, Vienna, Austria). Categorical variables were expressed as counts and percentages, and the chi-square test or Fisher’s exact test was used for comparison between groups. Continuous variables were presented as mean ± standard deviation and compared between groups using Student’s t-test (variables followed a normal distribution) or median and interquartile range (IQR) and compared between groups using a Wilcoxon test (variables did not follow normal distribution). A two-tailed p-value < 0.05 was considered to have statistical significance.

R software (R Foundation for Statistical Computing, Vienna, Austria, version 4.3.1) were applied for variable screening, nomogram model construction, and validation. In this study, 205 cases of candidemia patients from January 2006 to December 2023 were included as the training set. 29 cases of candidiasis patients from January to October 2024 were included as the validation set. Least absolute shrinkage and selection operator (LASSO) with ten-fold cross-validation and lambda 1 se as the criterion was applied to select the optimal combination of risk factors. Meanwhile, univariate and multivariate regression were applied to screen the valuable risk factors. A nomogram for predicting 30-day mortality was constructed based on the screening factors by the above two methods. The accuracy of the nomogram was evaluated by the area under the curve (AUC) the receiver operating characteristic (ROC) curve. To assess the difference between the predicted values from the nomogram and the actual values, a calibration curve and the Hosmer-Lemeshow goodness-of-fit test were used. Moreover, the clinical utility of the prediction model was evaluated through decision curve analysis (DCA).

**Figure 1:**
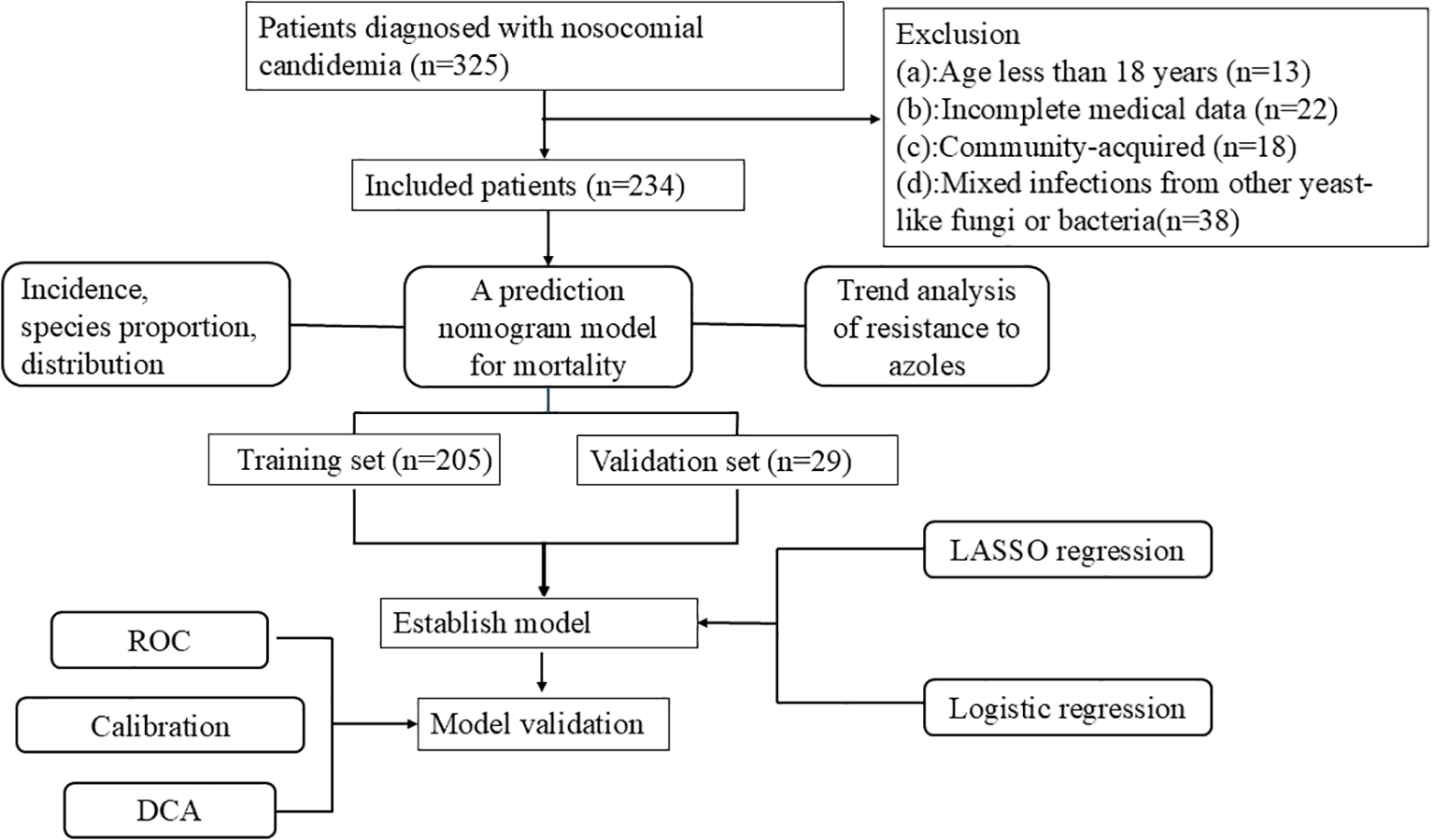
Flowchart for candidemia inpatients inclusion and exclusion.

Baseline description and statistic difference were automatically identified using the comparegroups package. LASSO regression was performed using the glmnet package. Multivariate logistic regression was performed using the glm package. Discriminatory analysis was performed using the ggROC package. Calibration was performed using rms package, DCA curves were performed using the rmda package and nomogram was constructed using the rms package. **Figure 1** showed the procedure of the study.

## 3 Results

### 3.1 Distribution of Candida species

**Figure 2:**
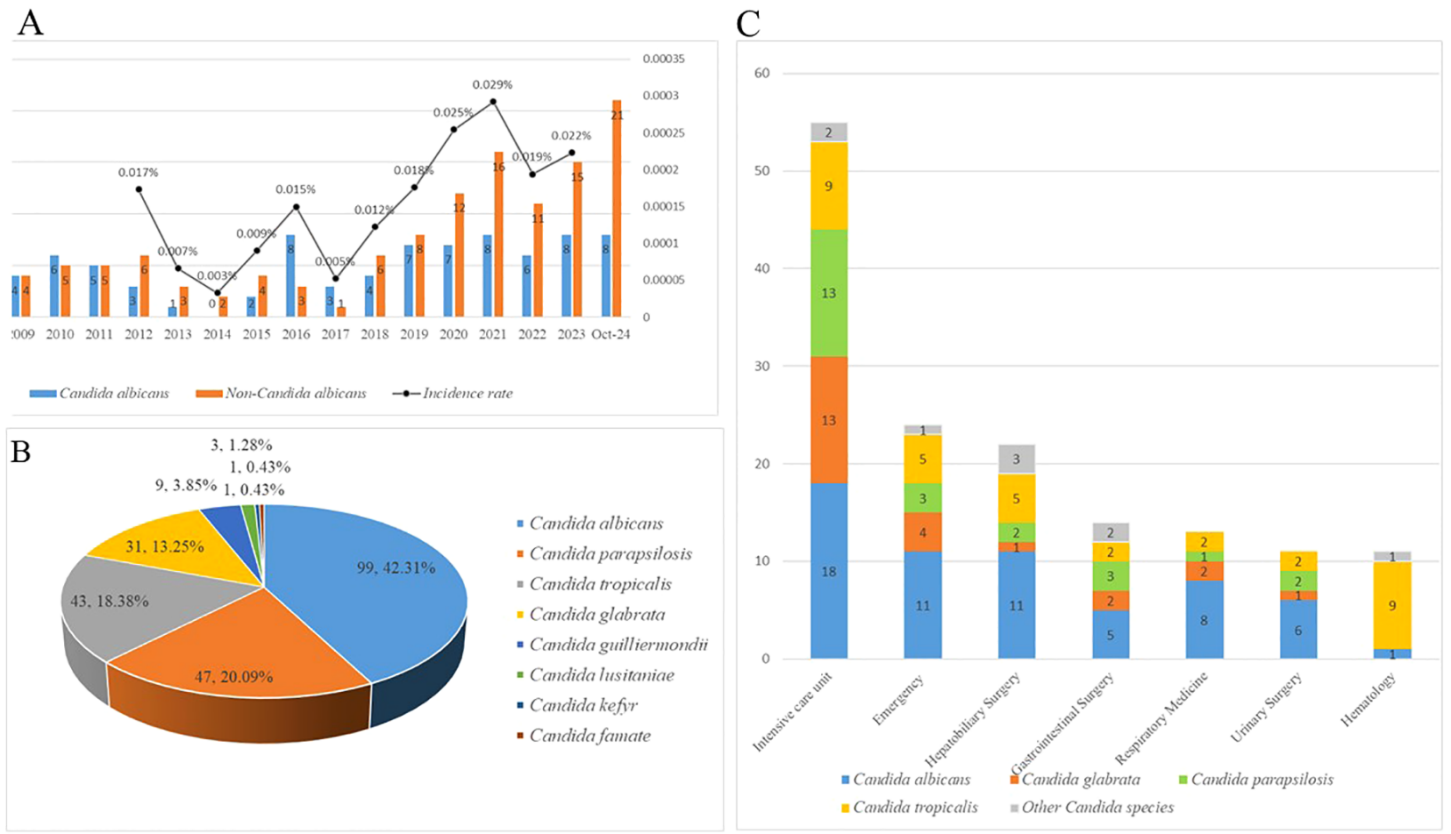
**(A)** The incidence rates of candidemia from 2021 to 2023. Due to the lack of total inpatient data from 2006 to 2012, and 2024 statistics only include candidemia patients from January to October. The incidence rate of candidemia patients in these years could not be calculated. **(B)** The proportion and the number of different *Candida species* of candidemia. **(C)** The proportion and the number of different *Candida species* of candidemia in different departments.

The incidence of candidemia has not displayed a directional trend, with higher rates recorded in the years 2020 (0.025%), 2021(0.029%), and 2023(0.022%) respectively. The number of candidemia with *non-albicans Candida* has obviously increased in recently five years (2020:n=12, 2021: n=16, 2022:n=11, 2023: n=15, 2024 January to October: n=21), as shown in **Figure 2A**. During the 19 years study, a total of 234 nosocomial candidemia patients were reported, containing a high proportion of *Candida albicans* (n=99,42.31%), followed by *Candida parapsilosis* (n=47, 20.09%), *Candida tropicalis* (n=43,18.38%), *Candida glabrata* (n=31, 13.25%) and *Candida guilliermondii* (n=9,3.85%), as shown in **Figure 2B**. Among the different hospital departments, a large number of cases were reported from Intensive Care Unit (n=55), followed by Emergency (n=24), Hepatobiliary Surgery(n=22), Gastrointestinal Surgery(n=14), Respiratory Medicine(n=13), Urinary Surgery (n=11), and Hematology(n=11). *Candida ablicans* was the predominant species in most departments, and *Candida tropicalis* was the dominant species in Hematology (9,81.81%), the proportional distribution of mainly *Candida* species in departments was shown in **Figure 2C**.

### 3.2 Antifungal resistance profiles

The antifungal resistance profiles of four mainly *Candida* species reported in the study were summarized in **Table 2**. Among the four mainly antifungal drugs, the highest susceptibility of were reported for amphotericin B, and only 1 case *Candida glabrata* resistant species against amphotericin B had been identified.

**Table 2:** Antifungal susceptibility of mainly Candida species in the current study.

Candida species	Antifungal drug	Tested organisms (n)	S/WT [n (%)] ^a^	SDD/I [n (%)] ^b^	R/non-WT [n (%)] ^c^
*Candida albicans*	Amphotericin B	99	99 (100.00)		
	Fluconazole	99	67 (67.68)		32 (32.32)
	Voriconazole	85	60 (70.59)	5 (5.88)	20 (23.53)
	Itraconazole	99	67 (67.68)	1 (1.01)	31 (31.31)
*Candida parapsilosis*	Amphotericin B	47	47 (100.00)		
	Fluconazole	47	39 (82.98)	3 (6.38)	5 (10.64)
	Voriconazole	39	30 (76.92)	6 (15.38)	3 (7.69)
	Itraconazole	47	40 (85.11)		7 (14.89)
*Candida tropicalis*	Amphotericin B	43	43 (100.00)		
	Fluconazole	43	19 (44.19)		24 (55.81)
	Voriconazole	40	16 (40.00)	2 (5.00)	22 (55.00)
	Itraconazole	43	18 (41.86)		25 (58.14)
*Candida glabrata*	Amphotericin B	31	30 (96.77)		1 (3.23)
	Fluconazole	31	1 (3.23)	29 (93.55)	1 (3.23)
	Voriconazole	31	17 (54.84)		14 (45.16)
	Itraconazole	31	29 (93.55)		2 (6.45)

^a^Susceptible or wild type; ^b^Susceptible dose dependent or intermediate; ^c^Resistant or non-wild type.

*Candida tropicalis* performed the highest resistance to azole (Fluconazole: 55.81%, Voriconazole:55.00% and Itraconazole:58.14%). The resistance of *Candida albicans* to fluconazole, voriconazole and itraconazole were 32.32%, 23.53% and 31.31%. *Candida parapsilosis* performed a lower resistance to azole (Fluconazole: 10.64%, Voriconazole:7.69% and Itraconazole:14.89%). Only one case fluconazole-resistant species of *Candida glabrata* were found.

### 3.3 Screening for predictive factors for 30-day mortality of candidemia

**Figure 3:**
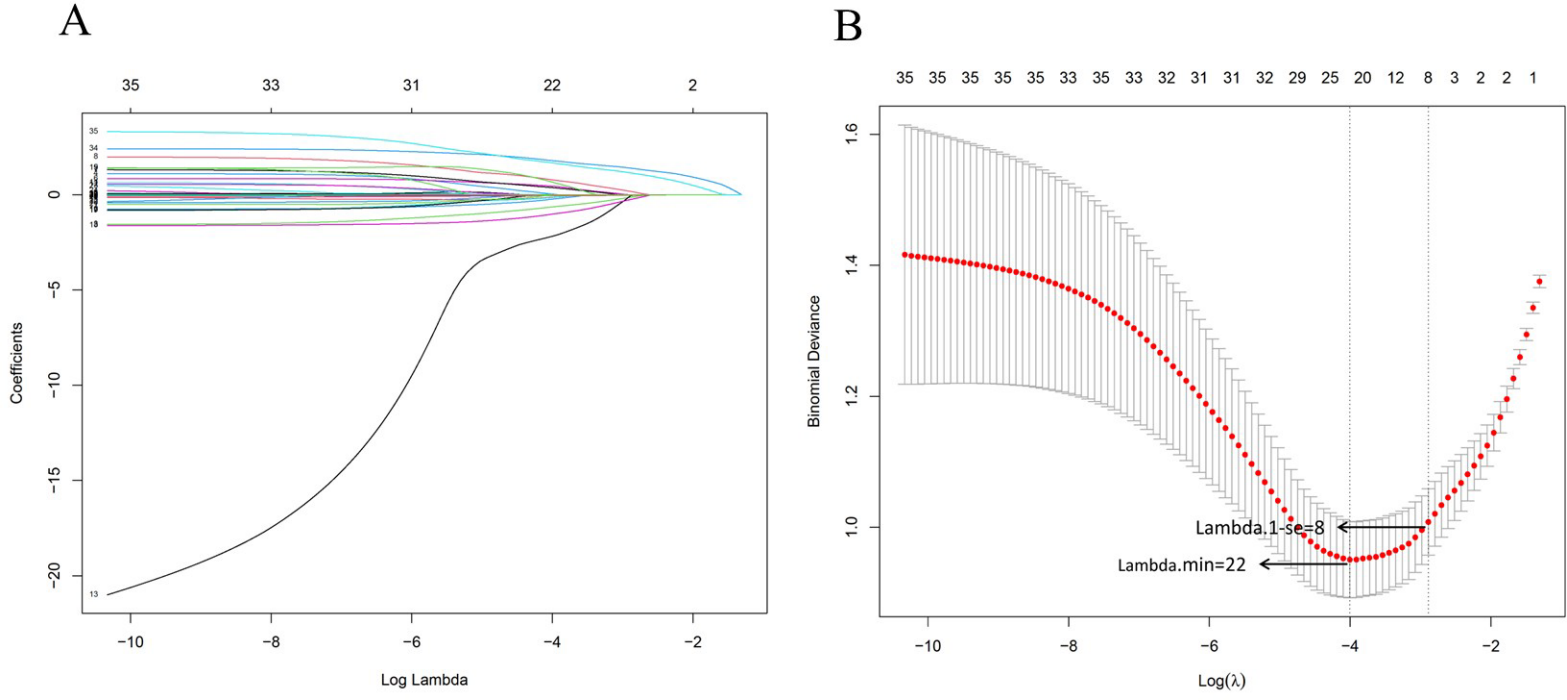
Variable selection using the least absolute shrinkage and selection operator and cross-validation. **(A)** LASSO feature selection was performed, with the horizontal axis representing the logarithm of the regularization parameter λ and the vertical axis representing the weights corresponding to each feature. As the λ increased, the feature weights gradually decreased until they reached zero and were excluded. **(B)** 10-fold cross-validation curve, with the vertical axis representing the mean squared error of the cross-validation. The λ value at the lowest point corresponds to the optimal regularization parameter, which balances the model complexity and predictive error. The Lambda.1min was 22 and the Lambda.1-se was 8, the lambda.1-se was used to screen the size of variables to structure the nomogram model.

According to the results of the 30-day follow-up after hospital discharge, the 30-day mortality rate of candidemia reached 52.99%. 35 independent variables were analyzed to determine the 30-day mortality of candidemia. Using the LASSO regression in training set to screen the diagnostic valuable variables based on lambda.min through 10-fold cross-validation. **Figure 3A** showed the LASSO feature selection, with the horizontal axis representing the logarithm of the regularization parameter λ and the vertical axis representing the weights corresponding to each feature. As λ increased, the feature weights gradually decreased until they reached 0 and were excluded. **Figure 3B** showed the 10-fold cross-validation curve, with the vertical axis representing the mean squared error of the cross-validation. The λ value at the lowest point corresponded to the optimal regularization parameter, which balanced the model complexity and predictive error. The “lambda.1se” in the results of the LASSO regression showed a model with optimal performance and the fewest size of independent variables. A total of 8 variables were included after selection via LASSO regression by lambda.1 se including *Candida albicans*, solid tumor, organ transplantation, urinary tract, albumin, hemoglobin, septic shock, multiple organ dysfunction syndrome were included to the risk scoring model:


$$\begin{array}{c} \text{Risk Score}=0.876\times Candida\;albicans+1.193\times \text{Solid tumor}\\ +0.945\times \text{Organ transplantation}+0.778\\ \times \text{Urinary tract}+0.969\times \text{Albumin}+0.999\\ \times \text{Hemoglobin}+4.049\times \text{Septic shock}+3.150\\ \times \text{Multiple organ dysfunction syndrome}. \end{array}$$

Simultaneously, all the 35 variables in training set were included in univariate and multivariate logistic regression, as shown in **Table 3**. Variables included solid tumor (OR=3.049 CI: 1.255-7.919 *P*=0.017), multiple organ dysfunction syndrome (OR=6.235 CI: 1.843-25.90 *P*=0.006), septic shock (OR=6.139 CI: 2.631-15.01 *P*<0.001),*Candida albicans* (OR=2.204 CI: 1.051-4.778 *P*=0.038) and albumin (OR=0.927 CI: 0.861-0.996 *P*=0.041) were considered to have statistical significance. Combining the two screening methods mentioned above, the variables including solid tumor, multiple organ dysfunction syndrome, septic shock, *Candida albicans* and albumin were applied to construct to nomogram model.

**Table 3:** Univariate and multivariate logistic regression analysis of the predictors for 30-day mortality of candidemia.

	Univariate analysis			Multivariate analysis		
	OR	95%CI	P	OR	95%CI	P
Age	1.021	1.007-1.036	0.005*	0.997	0.974-1.02	0.787
Sex/male	0.821	0.449-1.505	0.523			
Underlying conditions						
Hypertension	1.27	0.714-2.275	0.418			
Diabetes mellitus	1.523	0.783-3.038	0.222			
Chronic obstructive pulmonary disease	2.774	1.558-5.033	0.001*	1.686	0.753-3.808	0.204
Solid tumor	1.808	1.01-3.284	0.048*	3.049	1.255-7.919	0.017*
Hematologic malignancy	1.149	0.354-4.001	0.818			
Chronic renal failure	2.381	1.318-4.394	0.005*	0.763	0.309-1.831	0.549
Cerebrovascular disease ^a^	1.206	0.623-2.37	0.58			
Heart failure	3.304	1.612-7.244	0.002*	1.66	0.626-4.625	0.317
Cardiovascular disease ^b^	1.88	1.06-3.38	0.032*	1.265	0.513-3.139	0.609
Organ transplantation	0	NA-1.992	0.988			
Disseminated intravascular coagulation	0.811	0.096-6.865	0.836			
Rheumatism	0.813	0.032-20.73	0.884			
Multiple organ dysfunction syndrome	20.962	7.934-72.57	<0.001*	6.235	1.843-25.90	0.006*
Septic shock	11.804	6.198-23.41	<0.001*	6.139	2.631-15.01	<0.001*
Invasive procedures						
Central venous indwelling catheter	0.814	0.409-1.592	0.551			
Mechanical ventilation	1.374	0.788-2.411	0.265			
Gastrointestinal tract	1.182	0.682-2.055	0.551			
Urinary tract	0.614	0.341-1.093	0.1			
*Candida albicans*	2.049	1.169-3.633	0.013*	2.204	1.051-4.778	0.038*
Laboratory analytes						
White blood cell count (×10^9^/L)	1.049	1.004-1.098	0.035*	1.234	1.165-1.435	0.678
Neutrophil (×10^9^/L)	1.068	1.02-1.124	0.007*	1.385	1.278-1.656	0.203
Lymphocyte(×10^9^/L)	0.777	0.601-0.977	0.04*	0.904	0.782-1.024	0.139
Hemoglobin (g/L)	0.985	0.972-0.998	0.022*	0.989	0.972-1.006	0.209
Platelet (×10^9^/L)	1	0.999-1.002	0.517			
Albumin (g/L)	0.886	0.837-0.934	<0.001*	0.927	0.861-0.996	0.041*
Creatinine (μmol/L)	1.002	1-1.004	0.118			
Urea nitrogen (mmol/L)	1.064	1.029-1.105	0.001*	1.009	0.989-1.051	0.552
Alanine transaminase (U/L)	1.003	1-1.008	0.135			
Glutamic oxalacetic transaminase (U/L)	1.005	1.001-1.012	0.063			
Serum phosphorus (mmol/L)	1.027	0.479-2.204	0.945			
Platelet/Neutrophil	1.000	0.999-1.002	0.517			
Neutrophil/lymphocyte	1.091	1.037-1.157	0.002*	1.234	1.101-1.378	0.715
Platelet/lymphocyte	1.000	0.998-1.003	0.73			

*P<0.05 statistically significant.

### 3.4 Construct and validate the nomogram

**Figure 4:**
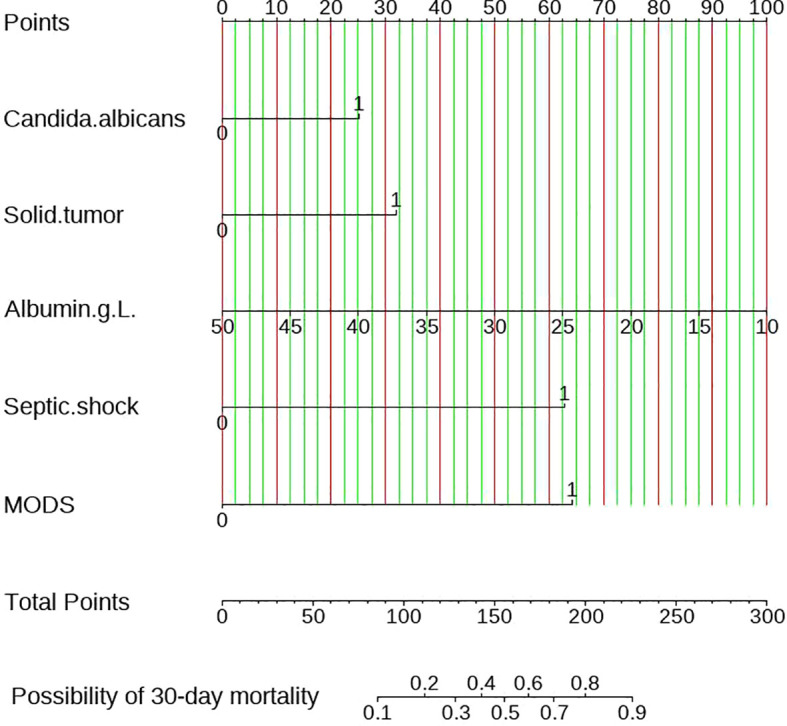
Construction of a nomogram model for the 30-day mortality rate of candidemia.

**Figure 4** represented the nomogram diagram, the scores of each independent predictor were on the upper scale, and the total score of five factors were shown in the lower scale. The total score correspond to the probability of 30-day mortality at the bottom of the graph. Taking a participant in this study as an example, this patient was infected with candidiasis with *candida albicans* and scored a 25 points on the scale. The serum albumin level was 30g/L, scored 50 points. The complications included multiple organ dysfunction syndrome and septic shock, earning scores of 64 and 62, respectively. The total score was 201 points, suggesting that the 30-day mortality was 81%. This finding was consistent with 30-day mortality of this patient.

**Figure 5:**
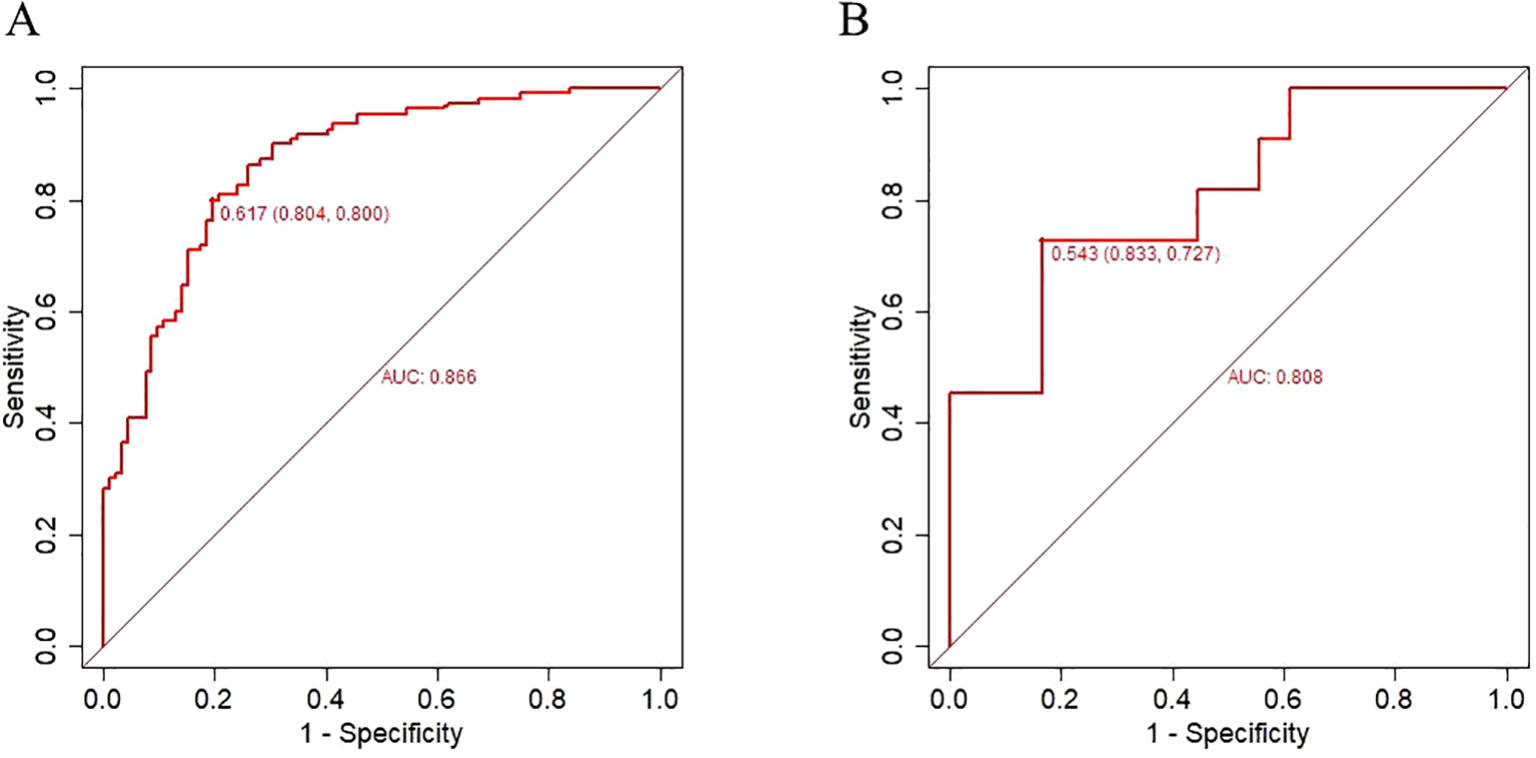
Receiver operating characteristic curve for evaluating the performance of nomogram for candidemia 30-day mortality. **(A)** training set **(B)** validation set.

**Figures 5A, B** analyzed the accuracy of the predictive model using the receiver operating characteristic (ROC) curve. The ROC curve intuitively demonstrates the model’s ability to distinguish between survivors and non-survivors after 30-day discharge. In the training set, the area under curve was 0.866 (95%CI: 0.817-0.916), the optimal cutoff value was 0.617, the sensitivity was 80.0% and the specificity was 80.4%.

In the validation set, the area under curve was 0.808 (95%CI:0.737-0.970), the optimal cutoff value was 0.543,The sensitivity was 72.7% and the specificity was 83.3%.

The results indicate that the nomogram developed had moderate predictive value (AUC > 0.7) and good discriminative ability. The Hosmer–Lemeshow test demonstrated an excellent agreement between prediction and observation (P=0.747).

**Figure 6:**
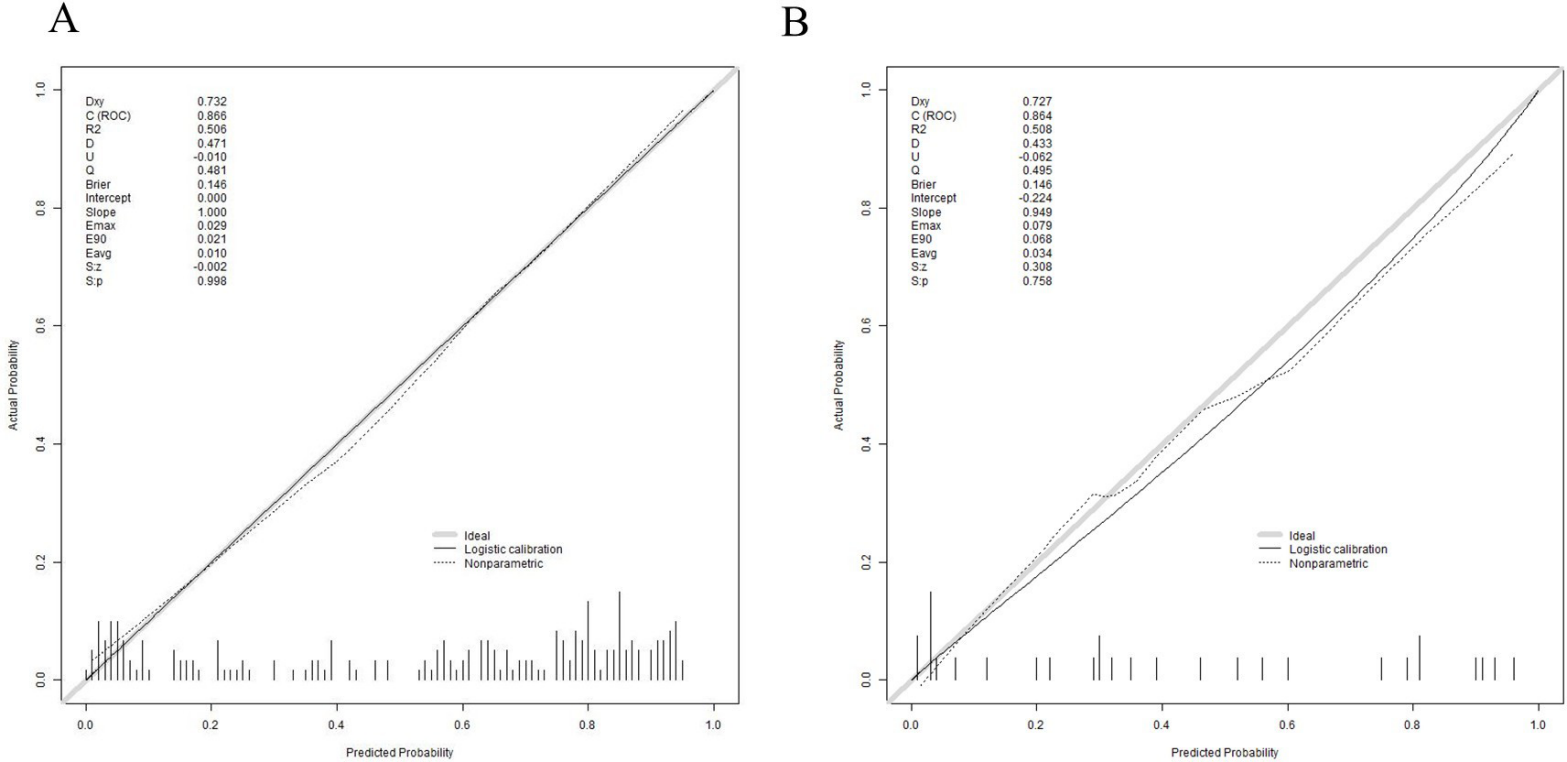
Calibration curve for validating the 30-day mortality nomogram model. **(A)** training set **(B)** validation set.

Subsequently, the Bootstrap resampling method was used for 1000 repetitions to create the calibration curve for the nomogram. The Apparent and Bias-corrected lines showed that the Brier score was 0.146 with a slope of 1 and P = 0.998 in the training set and the Brier score was 0.146 with a slope of 1 and P = 0.758 in the validation set. These results indicated that there were good consistency between the predicted probabilities and the actual occurrences, suggesting that the clinical prediction model has a certain degree of calibration (**Figures 6A, B**).

**Figure 7:**
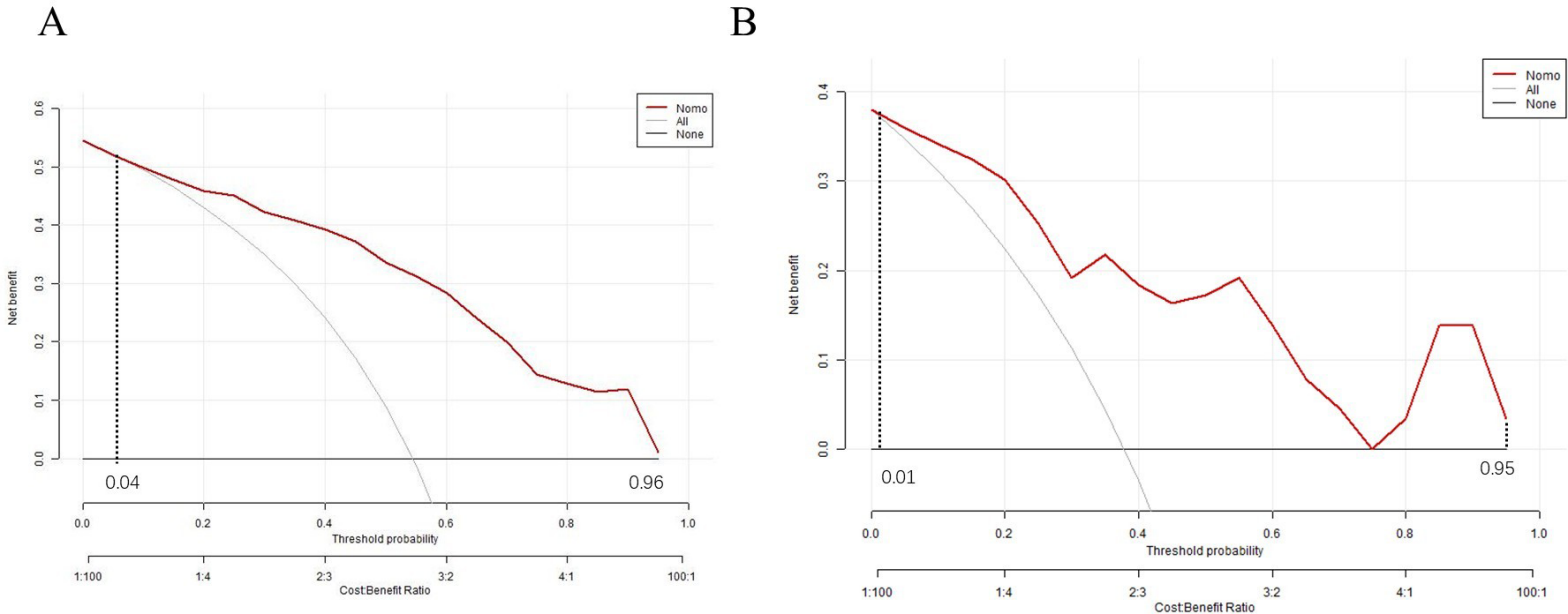
Decision curve of the nomogram model. **(A)** training set **(B)** validation set.

To evaluate the clinical utility of the nomogram, a decision curve analysis (DCA) curve was plotted. If the risk threshold was between 0.04 to 0.96,the nomogram will obtain net benefit in the training set, and if the risk threshold was between 0.01 to 0.95, the nomogram will obtain net benefit in the validation set. The curve showed that the net benefit was significantly higher than the two extreme values, as illustrated, indicating that the model has good clinical value (**Figures 7A, B**).

## 4 Discussion

In recent years, the incidence of nosocomial candidemia has gradually increased, especially among patients with compromised immune function. This study conducted a 19-year retrospective review on candidemia at a tertiary teaching hospital in southeastern coastal areas. The investigation covered demographic characteristics, underlying comorbidities, admitting departments, distribution of *Candida species*, antifungal drug resistance, and risk factors for mortality.

### 4.1 Epidemiology of candidemia

There was an increase in the incidence of candidiemia in recent years. The number of patients with *non-Candida albicans* infections obviously increased, which may be one of the main reasons for the rise in its incidence. Epidemiological trends for *Candia albicans* and *non-Candida albicans* differ significantly by region, diagnostic center, and patient type. *Candida albicans* remains the most common pathogen in candidemia. These findings are consistent with a report from a prospective observational study involving multiple centers in China (Pu et al., 2015).

Intensive Care Unit had the highest proportion of candidemia. Patients in the ICU commonly exhibit the following features including severe underlying illnesses, overuse of broad-spectrum antibiotics, and an increased frequency of invasive operations. All of these factors have increased the probability of candidemia (Rajni et al., 2022). Hepatobiliary Surgery is also a high-frequency department for candidemia. The reason may be that the surgeries performed in this department often involve liver transplantation or complex biliary reconstruction, which directly expose the organs, trachea, and vascular system, and these surgical procedures may lead to endogenous fungal infections. Emergency department is also a high-incidence department because it requires urgent treatment of trauma patients, frequent use of antibiotics and medical equipment, as well as timely management of various complications in patients (Chakrabarti et al., 2020).

It is worth noting that *Candida tropicalis* is the main causative agent of candidemia in the Hematology Department. A recent research in Algeria, neutropenia and leukemia were the most common underlying factors for *Candida tropicalis*-caused candidemia (Megri et al., 2020). The reasons for this phenomenon can be analyzed from the following two aspects. Firstly, most patients in the hematology are in an immunosuppression state, which makes them more susceptible to infections from *Candida species*. Furthermore, compared to other species of *Candida*, *Candida tropicalis* exhibits stronger pathogenicity and invasiveness, particularly in individuals with immunosuppression (Yang et al., 2023).

### 4.2 Analysis of antifungal drug resistance

The resistance rates to azole drugs vary among different *Candida* species. Regarding *Candida guilliermondii*, there is currently insufficient research to support the establishment of standard breakpoints, and assessments typically rely on clinical experience and results from *in vitro* susceptibility testing for individual evaluation. *Candida albicans* exhibit intermediate resistance to fluconazole, voriconazole, and itraconazole, which are slightly higher than in other parts of China (Pu et al., 2015). *Candida tropicalis* demonstrated the highest resistance rates to azole drugs compared with other candidemia species. This is consistent with reports from other parts of China (Yang et al., 2021; Ye et al., 2022). The high rates of azole resistance in *Candida tropicalis* are attributed to a combination of genetic alterations, overexpression of resistance genes, and environmental and cross-species transmission factors (You et al., 2017). *Candida albicans* has developed several resistance mechanisms against azole antifungals, including the overexpression of efflux pumps, mutations in the target enzyme Erg11, and biofilm formation, which collectively reduce the efficacy of treatment (Bosch et al., 2016). *Candida parasilosis* exhibited a relatively low resistance to azoles in this study, however, due to species distribution, local healthcare practices, antifungal usage, and environmental variables, the antifungal resistance of *Candida parapsilosis* varies greatly by area (Pfaller et al., 2011).

### 4.3 Nomogram model of 30-day mortality of candidemia patient

This study constructed a nomogram model for predicting the 30-day mortality of candidemia patients. The predictive model consists of five variables including *Candida albicans*, albumin, multiple organ dysfunction syndrome, solid tumor and septic shock. The nomogram model demonstrated excellent predictive performance and clinical utility.

The mortality rates associated with *Candida albicans* are generally higher compared to *non-Candida albicans*. Several mechanisms contribute to this increased mortality. *Candida albicans* candidemia are more frequent in patients with severe comorbidities, and undergoing invasive procedures. Multiple organ dysfunction syndrome and septic shock are significantly increases mortality in candidemia through several mechanisms. When pathogens enter the bloodstream, the immune system releases excessive cytokines causing systemic inflammation and this response can damage the body’s own tissues and organs (Candel et al., 2005). Septic shock leads to endothelial damage, increasing vascular permeability and causing fluid leakage into tissues. This results in hypovolemia, reduced cardiac output, and poor tissue perfusion, exacerbating organ dysfunction (Kang et al., 2010; Kwon et al., 2021). Persistent low blood pressure and poor perfusion result in multiple organ failures, including the kidneys, liver, heart, and lungs. Multiple organ dysfunction syndrome also is a leading cause of death in septic shock patients (Busani et al., 2017).

Solid tumors increase mortality in candidemia: the tumor and its treatments (e.g., chemotherapy, radiotherapy) impair the immune system, increasing vulnerability to *Candida* infections. Patients often experience neutropenia due to the tumor or its treatment, which increases the risk and severity of candidemia (Bergamasco et al., 2013). Albumin as a marker of nutritional status and overall health. Hypoalbuminemia often indicates poor nutritional status, which can weaken the immune system. Lower albumin levels can result from a cytokine-mediated acute-phase response to inflammation which are associated with higher mortality in infections (Santos et al., 2023).

## 5 Limitation

Despite the benefits described above, this study has inherent limitations. Firstly, the study used a single-center cross-sectional design and lacked validation with external data, if future prospective studies can be conducted across multiple centers, the effectiveness of the model can be further validated. Secondly, this study only analyzed the resistance of azoles and amphotericin B, more antifungal drugs such as echinocandins should be analyzed. Lastly, further research should focus on the molecular mechanisms of antifungal resistance in various *Candida species*.

## 6 Conclusion

The incidence of nosocomial candidemia has risen in recent years, with non-*albicans Candida* species increasing. *Candida albicans* is still the most common species of candidemia, but *Candida tropicalis* dominates in hematology. The incidence of candidemia was highest in the intensive care unit. *Candida tropicalis* exhibited significantly higher resistance rates to azoles. None of the above Candida species resistant to amphotericin B. A nomogram for predicting 30-day mortality after discharge with candidemia patients is constructed. Five independent risk factors included *Candida albicans*, multiple organ dysfunction syndrome, septic shock, solid tumors and decreased albumin.

## Data Availability

The raw data supporting the conclusions of this article will be made available by the authors, without undue reservation.
